# Development and Validation of a Semi-Automated, Preclinical, MRI-Template Based PET Image Data Analysis Tool for Rodents

**DOI:** 10.3389/fninf.2021.639643

**Published:** 2021-06-08

**Authors:** Fabian Schadt, Ina Israel, Samuel Samnick

**Affiliations:** Department of Nuclear Medicine, University Hospital Würzburg, Würzburg, Germany

**Keywords:** data analysis, Matlab, MRI, PET, positron emission tomography, preclinical imaging

## Abstract

**Aim:**

In PET imaging, the different types of radiotracers and accumulations, as well as the diversity of disease patterns, make the analysis of molecular imaging data acquired *in vivo* challenging. Here, we evaluate and validate a semi-automated MRI template-based data analysis tool that allows preclinical PET images to be aligned to a self-created PET template. Based on the user-defined volume-of-interest (VOI), image data can then be evaluated using three different semi-quantitative parameters: normalized activity, standardized uptake value, and uptake ratio.

**Materials and Methods:**

The nuclear medicine Data Processing Analysis tool (NU_DPA) was implemented in Matlab. Testing and validation of the tool was performed using two types of radiotracers in different kinds of stroke-related brain diseases in rat models. The radiotracers used are 2-[^18^F]fluoro-2-deoxyglucose ([^18^F]FDG), a metabolic tracer with symmetrical distribution in brain, and [^68^Ga]Ga-Fucoidan, a target-selective radioligand specifically binding to p-selectin. After manual image import, the NU_DPA tool automatically creates an averaged PET template out of the acquired PET images, to which all PET images are then aligned onto. The added MRI template-based information, resized to the lower PET resolution, defines the VOI and also allows a precise subdivision of the VOI into individual sub-regions. The aligned PET images can then be evaluated semi-quantitatively for all regions defined in the MRI atlas. In addition, a statistical analysis and evaluation of the semi-quantitative parameters can then be performed in the NU_DPA tool.

**Results:**

Using ischemic stroke data in Wistar rats as an example, the statistical analysis of the tool should be demonstrated. In this [^18^F]FDG-PET experiment, three different experimental states were compared: healthy control state, ischemic stroke without electrical stimulation, ischemic stroke with electrical stimulation. Thereby, statistical data evaluation using the NU_DPA tool showed that the glucose metabolism in a photothrombotic lesion can be influenced by electrical stimulation.

**Conclusion:**

Our NU_DPA tool allows a very flexible data evaluation of small animal PET data *in vivo* including statistical data evaluation. Using the radiotracers [^18^F]FDG and [^68^Ga]Ga-Fucoidan, it was shown that the semi-automatic MRI-template based data analysis of the NU_DPA tool is potentially suitable for both metabolic radiotracers as well as target-selective radiotracers.

## Introduction

Exact data evaluation in small animal studies is essential in diagnosing and treating diseases. Thereby, preclinical molecular imaging in neurological diseases using positron emission tomography (PET) remains challenging due to the wide variety of radiotracers and disease patterns as well as the lack of anatomical information.

Though there has been a variety of data analysis tools established, each tool has certain limitations. For example, the spmratIHEP Toolbox of Nie et al. (2014) is limited to [^18^F]FDG-PET images of rat brain. Amide’s a Medical Image Data Examiner (AMIDE) by Loening et al., again, requires a manual addition of a region of interest which is e.g., based on a non-organ shaped, geometrical ellipsoid (Loening and Gambhir, 2003). Although the Small Animal Molecular Imaging Toolbox (SAMIT) by Garcia et al. (2015) has been validated with data containing various radiolabeled tracers, its main focus lays on creating own PET and SPECT strain- and tracer-specific templates and less on data analysis. Additionally, the tool is limited by the restriction of the evaluation to the standardized uptake value (SUV) and the fact that there is no statistical evaluation available. Another frequently used program is the commercially available program PMOD (PMOD Technologies Ltd., Zurich, Switzerland). Beside the costs for the individual licenses, the program is limited to the application possibilities of the tool (including the given atlases). Adaptations to individual questions are difficult to fulfill without the possibility to adjust program code.

In this context, we wanted to implement and validate a Matlab-based nuclear medicine Data Processing Analysis tool (NU_DPA) using stroke-related rat brain PET data. The tool should not only be capable of processing the commonly used radiotracer [^18^F]FDG, but also be suitable for target-selective radiotracers. Therefore, the development of the tool will be based on PET datasets reflecting two types of radiotracers in two different kind of rat breeds (Sprague Dawley and Wistar). On the on hand, [^18^F]FDG, a metabolic radiotracer that is symmetrically distributed in brain (Fueger et al., 2006; Berti et al., 2014), and on the other hand the target-selective [^68^Ga]Ga-Fucoidan, which has a high affinity to bind specifically to p-selectin. P-selectin in turn is a protein, which is overexpressed primarily in the early phase after ischemic stroke (Okada et al., 1994; Israel et al., 2018).

Our goal was to develop a versatile and “compact” semi-automated data analysis tool that is capable to process and analyze data (of rodents) and that includes the following applications: First, the tool should be able to perform an automatic alignment of acquired PET images on a self-generated PET template for both types of radiotracers. Second, it should allow a detailed data quantification based on high-resolution MRI-based atlas information. And third, it should contain the possibility to also statistically evaluate the quantitative data including also non-parametric tests for small groups of data.

## Materials and Methods

### Animal Data

Functional PET data, which was used to implement and validate the tool, is based on PET datasets of (i) 14 male Sprague-Dawley rats (250–300 g, Harlan Winkelmann GmbH, Borchen, Germany), (ii) 10 adult male Wistar rats (250–300 g, Charles River Laboratories, Sulzfeld, Germany), and (iii) 14 adult male Wistar rats (250–300 g, Charles River Laboratories, Sulzfeld, Germany).

### Preparation of the Radiotracers

The applied 2-[^18^F]fluoro-2-deoxyglucose ([^18^F]FDG) was produced in-house at the Interdisciplinary PET-Centre (IPZ) of the University Hospital Würzburg using the GE-PETtrace cyclotron and the GE-Fastlab^®^ synthesis unit (GE Medical Systems, Uppsala, Sweden) as previously described (Israel et al., 2016; Lapa et al., 2016). A computer-assisted synthesis-module (Scintomics, Fürstenfeldbruck, Germany) was used for the synthesis of [^68^Ga]Ga-Fucoidan, as described previously (Li et al., 2014). Both radiotracers were manufactured under sterile conditions for clinical ([^18^F]FDG) and preclinical ([^68^Ga]Ga-Fucoidan) applications and were analyzed for radiochemical purity by HPLC and TLC before application.

### PET Acquisition

All measurements were performed on a mono-modality Siemens Inveon μPET scanner (Siemens Medical Solutions, Knoxville, United States). Animals were placed in prone position of the bed of μPET scanner. The alignment of the organ to be examined *in vivo* was performed manually, taking care to center it as best as possible in the field of view of the detector ring. During PET measurements, the animals were kept under anesthesia with a 2 % isoflurane in 100 % oxygen gas mixture and warmed with a heating pad to avoid hypothermia. The applied radiotracers were [^18^F]FDG for (i, ii) and [^68^Ga]Ga-Fucoidan for (iii). Injections were made via the tail vein. The average activities were (i) 31 ± 3 MBq, (ii) 43 ± 9 MBq and (iii) 26 ± 8 MBq. Data acquisition times were set from (i) 40–60 min, (ii) 0–60 min and (iii) 7–59 min after radiotracer application. Transmission scans (Isotope: Co-57) for data reconstruction were performed for 13 min after emission scans (i, ii) and for 6 min before emissions scans (iii), respectively. All PET datasets were reconstructed as dynamic scans directly after the emission and transmission scans using OSEM2D (i) or OSEM3D/MAP (ii, iii) of the scanner associated software package Inveon Acquisition Workplace (version 1.5.0.28). For reconstruction, default settings of the μPET scanner were used [OSEM2D: Sinogram Rebinning Algorithm = Fourier Rebin^∗^, Projection Filter = Ramp^∗^, Projection Cutoff (Nyquist) = 0.5, Iterations = 4, EM Iterations = 0; OSEM3D/MAP: Sinogram Rebinning Algorithm = None, Projection Filter = Ramp^∗^, Projection Cutoff (Nyquist) = 0.5, OSEM3D Iterations = 2, MAP Iterations = 18, Requested Resolution (mm) = 1.5] including attenuation correction from the transmission scans. The matrix size of the reconstructed images was 128 × 128 × 159 slices. The subdivision of the acquired data was done in 10 min frames (i, ii) and in 520 s frames (iii), respectively.

### Software Programs

The NU_DPA tool was implemented in the software program Matlab (version 2018a, MathWorks, United States). Beside some Matlab implemented toolboxes (Image Processing Toolbox, Statistics, and Machine Learning Toolbox), the following datasets, functions as well as toolboxes were implicated to test and validate the tool: (Table 1).

**TABLE 1:** Overview of the datasets, functions and toolboxes additionally included to the Matlab implemented toolboxes.

Name	Use
Sprague Dawley T2* and atlas MRI datasets (Papp et al., 2014) (i) Wistar T2* and atlas MRI datasets (Johnson et al., 2012) (ii, iii)	Anatomical sub-classification of the volume-of-interest
Export_fig (Altman, 2020)	Image high-resolution export
Ent (Agne, 2013)	Similarity measures
Medical Image Reader and Viewer (Schaefferkoetter, 2017)	Image visualization
Medical Image Registration Toolbox (MIRT) (Myronenko, 2010)	Affine (co-)registration
SWTest (Saïda, 2014)	Shapiro–Wilk normality test
Tools for NIfTI and ANALYZE image (Shen, 2020)	Import MRI data

The selection of the MRI datasets has been based on the focused organ of the examined rodent species (in our case the brain of the rat). The added MRI data and atlases were freely available and obtained from the indicated university sites mentioned in their manuscripts. For importing the MRI datasets, the *loadnii.m* function of the “Tools for NIfTI and ANALYZE image” is used (Shen, 2020). Registrations including affine transformations are performed with the MIRT algorithm, which is based on cubic B-splines parametrization (Rueckert et al., 1999; Myronenko, 2010). The MIRT algorithm includes various similarity measures, including Mutual Information, and is thus also suitable for co-registrations of datasets from different modalities. For “rigid” registrations (without scaling) the intensity-based image registration algorithm *imregister.m* implemented by Matlab is used. The *ent.m* and the *swtest.m* scripts are included for giving additional similarity measures information for a registration process (joint histogram, joint entropy, and mutual information) as well as for testing for normal distribution of the data (Agne, 2013; Saïda, 2014). The *VolumeViewer3D.m* script of the “Medical Image Reader and Viewer” allows manual visualization of 3D datasets in transversal, sagittal, and coronal sectional plane. If required, the images can be smoothed with a Gaussian filter for presentation (not applied to the images shown in this manuscript) (Schaefferkoetter, 2017). The script *Export_fig.m* allows saving graphics in high resolution (Altman, 2020). Graphics obtained in the data quantification and statistical analysis (see section “Data Quantification and Statistical Analysis”) are thereby automatically created and saved for all regions defined by the MRI atlas.

### NU_DPA Tool in Detail

**FIGURE 1:**
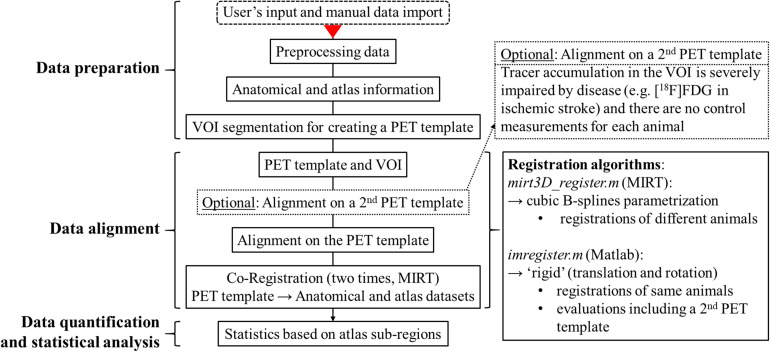
Flow chart of the structure and processing steps of the data analysis tool NU_DPA. The red arrow marks the processing step from which the NU_DPA tool runs automatically.

Figure 1 visualizes in form of a flow chart schematically the structure and processing steps of the NU_DPA tool. The red arrow marks the processing step from which the tool will work automatically. The listed processing steps that are performed by the NU_DPA tool are explained in more detail below.

#### Data Preparation

##### Preprocessing data

Before running the analysis, several preliminary inputs have to be given. These include for example input/output paths, time frames, experimental group or resolution parameters of the PET and the MRI datasets. Next, the reconstructed Siemens Inveon μPET data have to be imported into Matlab individually and manually. During this step, the first and last five peripheral slices are excluded to improve the signal-to-noise ratio. Furthermore, images are additionally transformed into a binary image in order to reduce small annihilation processes. With the help of the binary image, voxels with less than five connected components out of 26 possible can be located and eliminated from the non-binary images automatically. After import of all PET images, the program can be run.

In a first step, regarding future image processing steps and in order to obtain the highest intensity values, the time frames of each PET image are summed up. However, these “summed PET images” are just used for data alignment (see section “Data Alignment”) and not for data quantification and statistical analysis (see section “Data Quantification and Statistical Analysis”). Furthermore, the intensity differences of the “summed PET images” are adjusted by an individual multiplication factor within each assigned experimental groups. This step is necessary to compensate the variations of the applied radioactivity. The multiplication factor for each “summed PET image” is calculated by the ratio of the maximum averaged intensity value of all PET images to the averaged intensity value of the individual PET images. Additionally, based on input names and the assigned experimental group, the tool determines automatically whether multiple measurements have been taken for an animal (e.g., when examining the progression of a disease).

##### Anatomical and atlas information

**FIGURE 2:**
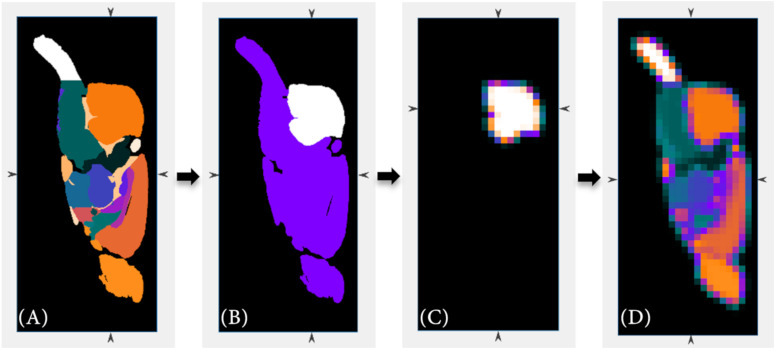
The high-resolution anatomical atlas **(A)** is resized to the lower resolution of the PET data **(D)** using Matlab’s *imresize3.m* function. Beside the full volume, the individual sub-regions of the anatomical atlas **(B)** are also resized **(C)** in order to allow a more detailed sub-classification (cerebellum region as example).

The volume-of-interest (VOI) is based on the added anatomical and atlas images. In our studies, the VOI corresponds with the brain of a rat. Based on the entered resolution parameters of the imaging modalities a scale factor is calculated to resize the added MRI T2^∗^ and atlas images to the lower resolution of the PET data using the *imresize3.m* function of Matlab. For the Sprague Dawley rat brain MRI dataset and atlas (Papp et al., 2014) and for the Wistar rat brain MRI dataset and atlas (Johnson et al., 2012), the scale factor is for example 1/20 and 1/31, respectively (PET resolution: 776 μm × 776 μm × 796 μm, MR resolution Sprague Dawley rat: 39 μm × 39 μm × 39 μm, MR resolution Wistar rat: 25 μm × 25 μm × 25 μm). The data are interpolated using the linear interpolation method. This allows a more detailed subdivision of the PET data. However, for this purpose, the individual sub-regions of the atlas must also be size-adjusted separately. Due to the known individual values of each sub-region, the percentage of each resized sub-region can then be calculated for each voxel. Regarding this, Figure 2 is to represent this adjustment step graphically once again.

##### VOI segmentation for creating a PET template

The VOI segmentation of the individual PET images in the experimental control group is used only to locate the PET image where the orientation of the VOI is best aligned along the axes. This PET image will then serve as the basis of the PET template. The automated detection of the VOI should therefore be as accurate as possible, but does not have to be perfect yet. The VOI selection is guided by comparing the number of connected components of the VOI in the PET image to the corresponding VOI in a (stereotaxic) MRI atlas using an adapting intensity-based threshold. Depending on the user’s input toward the applied radiotracer and whether the tracer accumulation is symmetrically distributed [e.g., [^18^F]FDG in brain) or target-selective (e.g., [^68^Ga]Ga-Fucoidan], the VOI segmentations proceed differently.

###### Metabolic radiotracer

**FIGURE 3:**
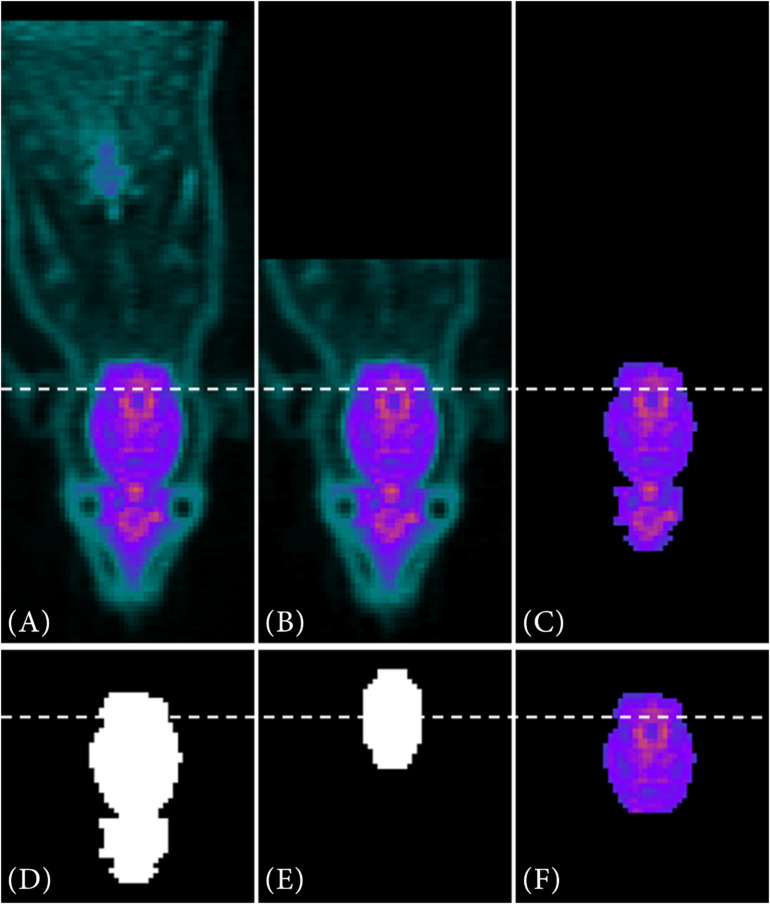
VOI segmentation steps for a metabolic radiotracer. In **(A)** the full PET image is depicted. After data reduction **(B)** and automatically threshold adaption using the Otsu method **(C)** a binary image is created out of the reduced data **(D)**. Via contour matching with the resized MRI atlas set as a binary image **(E)**, the VOI is segmented **(F)**.

The following Figure 3 shows exemplary on a PET image, how the VOI segmentation works for the metabolic radiotracer [^18^F]FDG.

Due to the manual placement and centering of the animal within the μPET scanner, the last third of the image data (Figure 3A) of the caudal region is deleted along the *z*-axis (Figure 3B). Next, by automatically adapting the image threshold using the Otsu method (*multithresh.m*), it reduces the data to a connected object, which is approximately the size of the resized MRI atlas in terms of their number of connected components (Figure 3C). Then, a binary image is generated from the reduced PET data (Figure 3D). Over this reduced PET data image, the resized MRI atlas (also set as binary mask, Figure 3E) is run stepwise and in 2° angle changes (−20°−20°) over it. By using contour matching (*bfscore.m*), the VOI is segmented (Figure 3F).

###### Target-selective radiotracer

**FIGURE 4:**
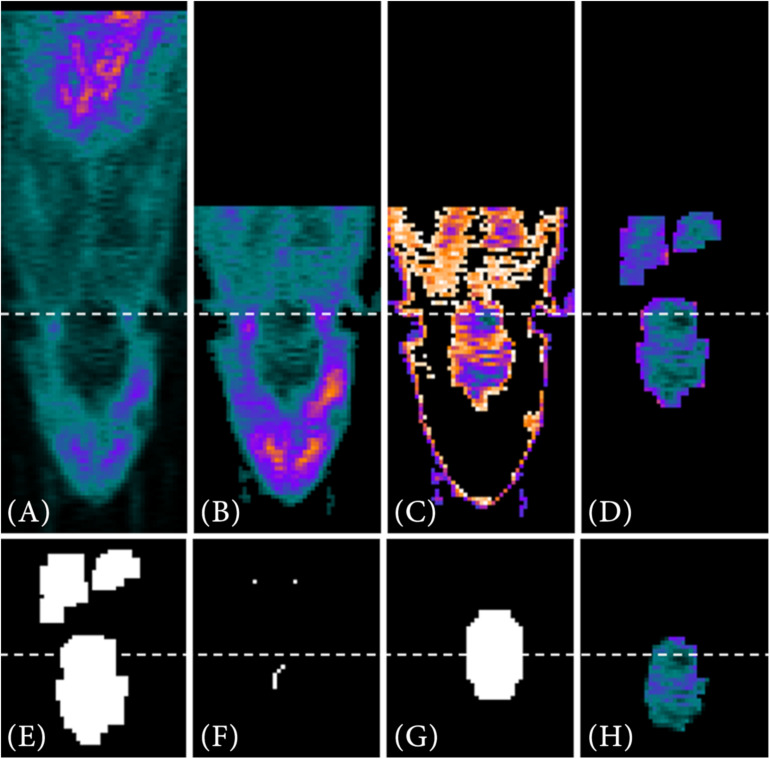
VOI segmentation steps for a target-selective radiotracer. First, the full PET image **(A)** is reduced using prior information **(B)**, such as the manual positioning of the small animal in the tomograph. Automatic threshold approximation using Otsu method **(C)** and pre-implemented Matlab morphological operations **(D)** further reduce the data. Next, a binary image is generated from the remaining PET data **(E)**. Using the lower resolution MRI atlas set as a binary image **(G)** and the interior voxels of the binary image as starting points **(F)**, the image is searched for the VOI via contour matching. In **(H)** the segmented VOI is shown as result.

For a target-selective radiotracer such as [^68^Ga]Ga-Fucoidan, the process of the VOI segmentation to determine the reference image of the PET template is shown exemplary on one PET image in Figure 4.

The first two steps of data reduction are comparable to the steps already described in the VOI segmentation of the metabolic radiotracer [^18^F]FDG (see section “Metabolic Radiotracer”). This includes the exclusion of the last third of image information in *z*-direction (Figure 4B) as well as the search for objects similar size to that of the resized MRI atlas using *multithresh.m* (Figure 4C). However, due to the low radiotracer uptake within the VOI, a sufficient VOI segmentation is difficult since also other structures contain a similar intensity values. An example for that would be [^68^Ga]Ga-Fucoidan, which targets p-selectin. Therefore, a (higher) tracer accumulation is expected specifically at the expressed p-selectin within the VOI, but not in the whole VOI itself.

Nevertheless, since the VOI is located within the animal and defined by specific volume, the image information can be further reduced using pre-implemented Matlab morphological operations (*bwconncomp.m*, *bwmorph3.m*, *imdilate.m*, *imerode.m*, and *watershed.m*) (Figures 4D–F). The remaining (former interior) voxels in Figure 4F then form the starting points for contour matching (*bfscore.m*) of the binary image in Figure 4E with the resized MRI atlas. For this, the resized MRI atlas is also set as binary image (Figure 4G). Since the location of the VOI in 3D space is not known, contour matching is performed for different orientations and angles of the resized MRI atlas (*imrotate3.m*, 2° angle changes from −20°−20°). Figure 4H shows the result for the automatically segmented VOI.

#### Data Alignment

##### General alignment information

All (co-)registration processes are performed using normalized data in order to compensate value differences. However, the actual alignment of the data is done using the original image data. Both registration algorithms used in NU_DPA, *mirt3D_register.m* (MIRT) and *imregister.m* (Matlab), additionally generate a transformation matrix at each registration. Such a transformation matrix includes the individual adjustment steps of the (co-)registration and can also be applied to further (aligned) images. For example, all the PET images already aligned on the PET template are aligned on the MRI atlas using the resulting transformation matrices of the two times affine co-registrations (see section “Co-registration”).

##### PET template and VOI

###### Creating the reference image

After segmentation of the VOI in all PET images in the experimental control group, the PET image to be used as reference for the PET template is determined. For this purpose, the angular orientations of the VOI in 3D space have to be calculated first. Decisive for the calculation of the angles are the VOI enclosing cuboid, the eigenvectors as well as the axis lengths, which can be obtained using the Matlab function *regionprops3.m*. Due to the positioning of the small animal in the tomograph and the specific ellipsoidal shape of the brain, the axis lengths are assigned from long to short to the orientations in *z*-, *x*-, and *y*-direction. The PET image that is best aligned to the axis directions (smallest angles in *z*-, *x*-, and *y*-direction) forms the reference image of the PET template on which all the other PET images of the experimental control groups are then registered onto. Before registration, the reference image is still centered in the 3D space using the centroid coordinates of the VOI also obtained by *regionprops3.m*.

###### PET template creation

The algorithm underlying the PET template creation of the experimental control group is the MIRT registration algorithm (Myronenko, 2010). MIRT performs non-rigid registrations of two 3D images using cubic B-spline based transformation parametrization. Since, in our case, only functional data has been processed, the similarity measure of the MIRT algorithm has been set to “correlation coefficient” (1,000 iteration steps, tolerance criteria 1e^–8^).

**FIGURE 5:**
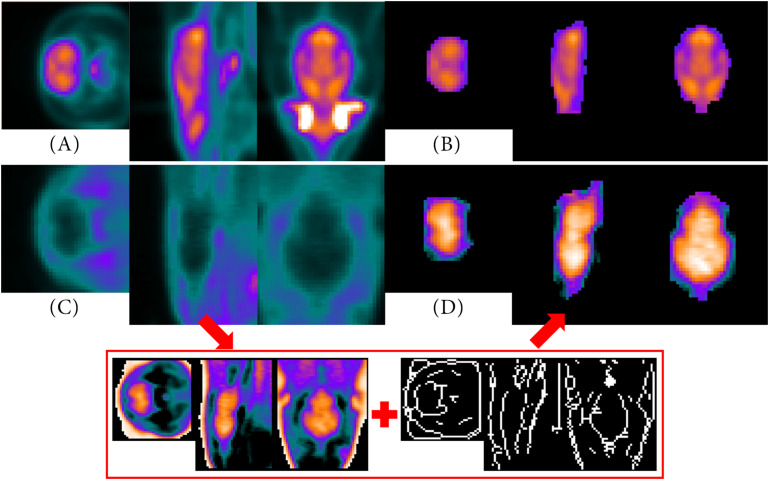
PET templates and segmented VOI. Images **(A,B)** are based on (i) seven sham-operated Sprague Dawley rats including [^18^F]FDG as applied radiotracer. **(C)** Represents a PET template averaged over (iii) 14 Wistar rats with a stroke lesion (applied radiotracer: [^68^Ga]Ga-Fucoidan). Using intensity inversion and the skeletonized gradient image (red box), the VOI can be segmented **(D)**. Since [^68^Ga]Ga-Fucoidan is a target-specific accumulating radiotracer, there is only low uptake in other regions of the VOI than the target itself, e.g., in the “healthy tissue” of the brain. However, due to the intensity adjustments and inversion, it appears that the larger “healthy tissue” of the brain shows a high radiotracer uptake, while the smaller region, which was targeted by the radiotracer, presents almost no accumulation.

Figure 5 visualizes as examples two PET templates created by the NU_DPA tool including the metabolic radiotracer [^18^F]FDG (Figure 5A) and the target-selective radiotracer [^68^Ga]Ga-Fucoidan (Figure 5C). Here, the PET template in Figure 5A is based on (i) seven sham-operated Sprague Dawley rats, while the PET template in Figure 5C is the averaged image of (iii) 14 Wistar rats with a stroke lesion. For future co-registration with the MRI T2^∗^ image, the VOI of the PET template is additionally segmented. Thereby, the VOI segmentation of the PET template for the metabolic radiotracer [^18^F]FDG (Figure 5B) is similar to that described in section “Metabolic Radiotracer.”

However, the low intensities within the “healthy brain tissue” of the VOI of the [^68^Ga]Ga-Fucoidan PET template make it difficult to segment the VOI as accurately as possible. Therefore, VOI segmentation needs to be optimized compared to the descriptions in section “Target-Selective Radiotracer.” First, the image is enclosed by a cuboid to reduce data information. This is possible since the VOI of the reference image has been centered before PET template creation. Due to the target-specificity of the radiotracer, in this case the high binding in the stroke infarct, a (high) radiotracer accumulation in the VOI itself is not assumed. By excluding the intensities with higher values than half of the maximum intensity, the data therefore can be adequately reduced. Due to the fact that the remaining data now contains low intensity values, the image intensity values are adjusted (*imadjust.m*) and inversed to improve identifying the “healthy brain tissue”. This means, high values become low values and vice versa [Figure 5 (red box, left)]. Using pre-implemented Matlab morphological operations (e.g., *imdilate.m*), a single voxel positioned in the center of the 3D space is gradually dilated to the size of the VOI (binary mask). The skeletonized gradient image [Figure 5 (red box, right)] of the normalized and intensity values inversed image [Figure 5 (red box, left)] serves as a boundary of the size. The result of the VOI segmentation after multiplying the “dilated binary mask” with the normalized and intensity values inversed image [Figure 5 (red box, left)], is shown in Figure 5D.

##### Alignment on the PET template

Based on the user’s input, images containing same name designations but different experimental states (e.g., name: “Rat_No_1,” state: “healthy” vs. name: “Rat_No_1,” state: “stroke”) are registered onto each other. The used registration algorithm is the *imregister.m* algorithm (“rigid”) implemented by Matlab (2000 iteration steps, tolerance criteria 1e^–21^). If a control measurement of the same animal is available, the “rigid” registration is performed from the disease state(s) to the control state. Unfortunately, “rigid” registration processes can easily mismatch by a falsely rotation or shifting, e.g., when irrelevant areas such as the animals’ paws influence the alignment. Even when focusing on registering two VOI alone, the alignment often does not match due to rotational errors. Therefore, additional structures, which are linked to the actual VOI are taken into account for registration.

For the metabolic radiotracer [^18^F]FDG for example, the Harderian glands of the rat are added to the VOI. These Harderian glands usually have a high [^18^F]FDG uptake, which is not strongly affected, even by a change of radiotracer accumulation within the VOI (e.g., after ischemic stroke). The reduction of the data is carried out according to the explanations in section “Metabolic Radiotracer.” However, the process already ends after the volume approximation via threshold. A VOI including the Harderian glands as additional structures is similar to the representation in Figure 3C.

**FIGURE 6:**
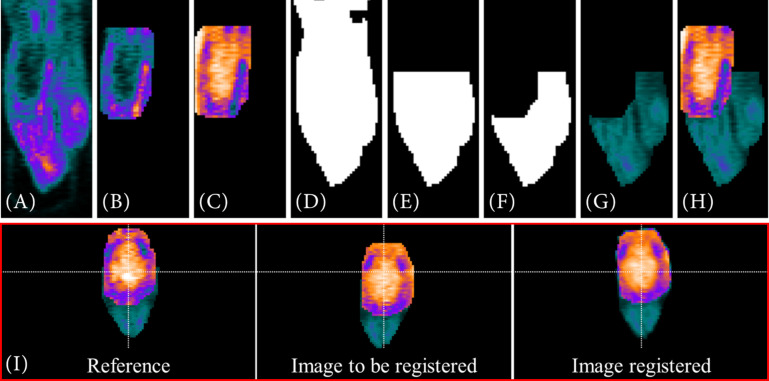
Visualization of a “rigid” registration of two [^68^Ga]Ga-Fucoidan images including the rat’s head for optimizing the registration process. **(A–H)** A description of how the head of the rat is added to the intensity-inverse VOI. Both images, reference and image to be registered, are based on a combination of intensity-inversed, segmented VOI **(B,C)** and rat’s head **(G)**. To extract the head, the image is transformed into a binary image **(D)**. The binary image is then reduced using the enclosing cuboid of the VOI obtained with *regionprops3.m* **(E)**. Next, the voxels already included in the VOI are excluded **(F)** before multiplying the reduced binary image as mask with the initial image **(A)**. At last, the intensity-inversed VOI **(C)** and the extracted head **(G)** are combined. **(H)** The result of such a VOI/addition structure combination. The lower images (**I**, red box) show the result of such a “rigid” registration.

Target-selective radiotracers do usually not have such highly tracer-accumulated structures connected to the VOI. Nevertheless, in order to achieve a suitable alignment of the data, the rat’s head is added to the intensity-inversed VOI. For this purpose, the segmented VOI (see section “Target-Selective Radiotracer”) is extracted from the original image (Figure 6A) in a dilated form (*imdilate.m*, Figure 6B). Next, similar to the VOI segmentation of the PET template (see section “PET Template and VOI”), the intensity values are inversed. Thus, the low tracer accumulation in the “healthy brain tissue” is highlighted (Figure 6C). In order to add the head of the rat, a filled, binary image is created out of the image (Figure 6D). This binary image is reduced in *x*- and *y*- direction to the dimensions of the enclosing cuboid of the VOI (*regionprops3.m*) and in *z*-direction to the first half (Figure 6E). Furthermore, all voxels already included in the intensity-inversed VOI are excluded Figure 6F. The initial image is then multiplied by the created binary mask and its intensity values are halved since the head of the rat should remain a “helping” structure (Figure 6G). In a final step, intensity-inversed VOI and the head of the rat are combined (Figure 6H). The images in Figure 6I (red box) show an example for this type of data alignment.

Registrations including different animals are performed using MIRT (Myronenko, 2010). This is generally the case when the images of the control group are registered onto the PET template.

##### Alignment on a 2nd PET template

However, if the tracer accumulation between the experimental and the control group might alter severely, data evaluation can be performed by including a 2nd PET template. An example for such a severely alteration would be the [^18^F]FDG accumulation after an ischemic stroke. A 2nd PET template is intended to compensate different manifestations of the disease and to mitigate effects caused by strong inflammatory processes or the surgical procedure. Whether an evaluation is made with the help of a 2nd PET template depends on the initial input of the user. If it seems appropriate, the 2nd PET template should be based on the experimental group that has the least discrepancy with the control group images. A 2nd PET template can also be particularly useful when, e.g., control group and experimental group do not contain the same animals or the same number of animals.

Volume-of-interest segmentation for creating a 2nd PET template follows the description listed in section Metabolic Radiotracer.” Reference image for the 2nd PET template is determined as described previously in section “PET Template and VOI.” However, even though the data contains different animals, data alignment is done using the *imregister.m* algorithm (“rigid”) including additional structures (see section “Alignment on the PET Template”). Inflammatory processes and the strongly altered tracer accumulation would otherwise influence the MIRT registration algorithm, which then would result in insufficient and false alignments. All PET images aligned to the 2nd PET template would then be aligned to a comparable PET template, also using the “rigid” registration.

##### Co-registration

**FIGURE 7:**
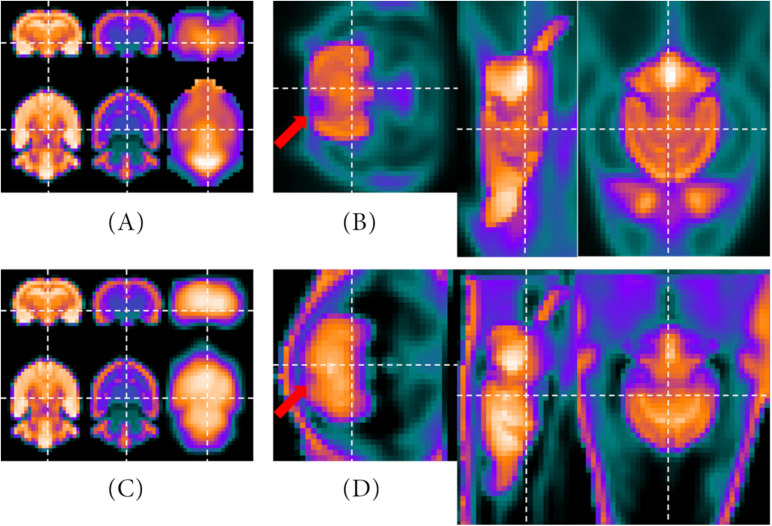
Results of the co-registration processes. The upper row represents the optional 2nd PET template which is averaged out of (ii) 10 Wistar rats with ischemic stroke ([^18^F]FDG) aligned with the representative MRI images. On the left side, the MRI T2* and atlas of the Wistar rat brain (Johnson et al., 2012) as well as the VOI of the optional 2nd PET template are presented **(A)**. On the right side, the MRI atlas is included in the image of the optional 2nd PET template **(B)**. The bottom row shows the same images **(C,D)**, but with the [^68^Ga]Ga-Fucoidan data [(iii) 14 Wistar rats with stroke lesion] instead of the optional 2nd PET template. Here, the VOI is shown in the intensity-inversed representation, whereas the PET template is not. The red arrows mark the regions affected by the stroke.

As the last step before statistical data analysis, the PET template is co-registered in two steps with affine alignments to the MRI T2^∗^ image using the MIRT algorithm. Thereby, the first registration is a pre-adjustment step, roughly aligning both images by their gradient images, similar to Gianpaolo et al. (2010). The second registration is based on the segmented VOI of the PET template (Figures 5B,D). Similarity measure is set to “mutual information” (500/2,000 iteration steps, 1e^–15^ tolerance criteria for both). Since the atlas information correspond with the MRI T2^∗^ image, the process of co-registration is equivalent to alignment on the atlas. Figure 7 shows two examples of co-registered PET templates. Both studies have contained Wistar rats, which is why the PET templates have been aligned to the resized MRI atlas of Johnson et al. (2012).

The images in the upper row show the results of the co-registration with the optional 2nd PET template containing (ii) 10 Wistar rats with ischemic stroke ([^18^F]FDG). On the left side (Figure 7A), the MRI T2^∗^, the MRI atlas and the PET template VOI are presented beside each other. On the right side (Figure 7B), the MRI atlas is shown within the 2^*nd*^ PET template. The tracer accumulation which was affected by the ischemic stroke is marked by the red arrow. For the images in the lower row, instead of the data of the 2^*nd*^ PET template, the intensity-inversed “healthy brain tissue” of the (iii) 14 Wistar rats with stroke lesion ([^68^Ga]Ga-Fucoidan) is presented in the left images (Figure 7C). The right image shows the matched MRI atlas and PET template of the target-selective radiotracer [^68^Ga]Ga-Fucoidan (Figure 7D). Here, the red arrow marks the stroke lesion.

Various similarity measures are included to track how accurately a registration process occurred. These include for example the correlation coefficients according to Pearson and Spearman as well as the joint entropy and mutual information [*ent.m*, (Agne, 2013)]. Similarity measures are calculated before and after (co-)registration processes between the voxel values of two full images and VOI, respectively. Classifications can then help to assess the accuracy of the registration. For example, Schober et al. (2018) have given a conventional approach for the interpretation of the correlation coefficient. Nevertheless, all registration processes should still be verified visually.

#### Data Quantification and Statistical Analysis

Data quantification and statistical analysis is performed using the individual sub-regions of the MRI atlas information on which the data are aligned (in brain, e.g., thalamus, cerebellum, etc.). Thereby, three semi-quantitative analysis parameters are implemented in the tool for analyzing the data, equivalent to Miederer et al. (2018). These parameters are the normalized activity (A_*N*_), the standardized uptake value (SUV) and the uptake ratio [UR, also referred to standardized uptake value ratio, SUVR (Schwarz et al., 2017)].

$$\begin{array}{c} A_{N}=\frac{measuredradioactivityinasubregionoftheVOI}{injectedradioactivity}\\ SUV=\frac{radiotracerconcentrationinasubregionoftheVOI}{\frac{injectedradioactivity}{bodyweight}}\\ UR=\frac{measuredradioactivityinasubregionoftheVOI}{\begin{array}{c} measuredradioactivityinthewholeVOI\;\\ orreferencessubregion\; \end{array}} \end{array}$$

After the calculation of the semi-quantitative analysis parameters, the tool outputs boxplots including individual values of each animal for each sub-region of the atlas. Thus, the measured data can be visually verified and also checked for possible outliers. Numerical tests like Anderson-Darling and Shapiro–Wilk as well as QQ-plots for visual illustration are added as tests for normal distribution of the individual VOI sub-regions. The plots (boxplot and QQ-plots) are also automatically generated and saved in high-resolution by the NU_DPA tool [*Export_fig.m*, (Altman, 2020)]. Basis of the graphs are the Matlab-implemented algorithms *boxplot.m* and *qqplot.m*.

For the calculated semi-quantitative results, the tool offers various statistical tests to check for significant differences between the experimental groups. Based on the number of images per group and whether the groups are paired or unpaired, the tool chooses either nonparametric (*n* < 30) or parametric (*n* ≥ 30) tests, like for example Wilcoxon rank sum test (two experimental groups) or Friedman test (>two experimental groups, *post hoc*: Wilcoxon rank sum test) for nonparametric, paired groups.

## Results

The following data show an example of the result of a three-way comparison between three different experimental states. The data is part of (ii) 10 adult male Wistar rats, of which six adult male Wistar rats were also measured in the healthy control state. One day after PET measurement in the healthy control state, a photothrombosis (PT) was induced in the right sensorimotor cortex in each of the six animals. In addition, a microelectrode was implanted in the right mesencephalic locomotor region. A three-way comparison was performed between the six adult male Wistar rats for the following different experimental states: healthy control state (named Sham), induced photothrombosis and microelectrode but no deep brain stimulation (PT unstimulated, 3 days after PET measurement in the healthy control state), and induced photothrombosis and microelectrode including deep brain stimulation (PT stimulated, 4 days after PET measurement in the healthy control state). The aim was to analyze the [^18^F]FDG accumulation for each experimental state with focus on the right sensorimotor cortex affected by ischemic stroke. Since the underlying Wistar MRI atlas (Johnson et al., 2012) only includes the cortex as one large region, the cortex had to be split manually into left and right hemispheres for differentiation.

Data alignment of each animal was performed by registering both PT images (PT unstimulated and PT stimulated) on the Sham image. Registration was performed using the *imregister.m* registration algorithm (“rigid”), with focusing on the VOI including the Harderian glands as additional structures. The Sham PET images were then aligned with the PET template, created from the six Wistar rats in the healthy control state, using the *mirt3D_register.m* (MIRT). The already aligned PET images, PT unstimulated and PT stimulated, were then transformed by using the created transformation matrix. Pearson’s correlation coefficient was 0.96 ± 0.01 for the PET template calculated for the aligned PET images after registration with the reference image. Finally, the PET template was co-registered in two steps with affine alignments to with the Wistar MRI T2^∗^-weighted image (Johnson et al., 2012). The relevant Spearman correlation coefficient was 0.82.

Prior to data quantification and statistical analysis, the alignments of the VOI of the PET images were visually verified slice by slice for each animal and image. For this purpose, the data were displayed side by side using the pre-implemented Matlab function *circshift.m* and *VolumeViewer3D.m*. Due to the surgical intervention and the implanted microelectrode, the UR for each region of the atlas was not calculated over the measured radioactivity in the whole brain but the measured radioactivity in the cerebellum.

$$UR=\frac{measuredradioactivityinasubregionoftheVOI}{measuredradioactivityintheCerebellum}$$

The cerebellum was chosen as reference region since it was not affected by the operational process as well as since it did not show significant differences in the [^18^F]FDG uptake within the cerebellum of similarly comparable neurological studies (Putzu et al., 2018; Wang et al., 2020). In consequence of the low number of PET images per group, the three-way comparison of the UR was statistically analyzed for each region using the non-parametric friedman test (*friedman.m*) provided by Matlab. *Post hoc* test was the Wilcoxon signed rank test (*signrank.m*). Table 2 shows the results of the *post hoc* analysis for the five largest atlas regions of the Wistar rat brain atlas (Johnson et al., 2012), including the cortex region, manually divided into left and right hemispheres.

**TABLE 2:** Results for the five largest atlas regions of the Wistar MRI atlas (Johnson et al., 2012) of the three-way comparison between healthy control state (Sham), photothrombosis without deep brain stimulation (PT unstimulated, PTu) and photothrombosis including deep brain stimulation (PT stimulated, PTs).

No.	Region	Experimental groups	Time interval	*p*-value
1	Isocortex	PTu–PTs	0–20 min	–
			20–60 min	*
		PTu–Sham	0–60 min	*
		PTs–Sham	0–60 min	*
	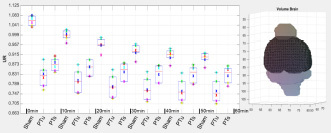			
	Isocortex left	PTu–PTs	0–20 min	–
			20–60 min	*
		PTu–Sham	0–60 min	*
		PTs–Sham	0–60 min	*
	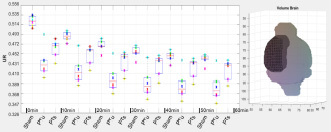			
	Isocortex right	PTu–PTs	0–20 min	–
			20–60 min	*
		PTu–Sham	0–60 min	*
		PTs–Sham	0–60 min	*
	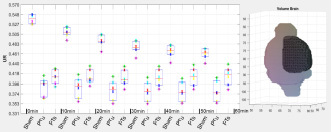			
2	Olfactory structures	PTu–PTs	20–60 min	–
		PTu–Sham	20–60 min	*
		PTs–Sham	20–60 min	–
3	Corpus callosum	PTu–PTs	0–30 min	–
			30–60 min	*
		PTu–Sham	0–60 min	*
		PTs–Sham	0–60 min	*
4	Hippocampal formation	PTu–PTs	0–60 min	–
		PTu–Sham		*
		PTs–Sham		*
5	Striatum	PTu–PTs	0–60 min	–
		PTu–Sham		*
		PTs–Sham		*

PTu, PT unstimulated; PTs, PT stimulated; *p ≤ 0.05; –, not significant.

The results showed that the uptake of [^18^F]FDG in the right sensorimotor cortex affected by ischemic stroke can be significantly influenced by specific deep brain stimulation.

## Discussion

The aim of creating the NU_DPA tool was to develop a “compact” semi-automated data analysis tool, which should offer the possibility to analyze imaging data of rodents for various types of radiotracers. In our studies, we were able to show that the tool is capable of processing radiotracers with symmetrically distributed uptake in brain [e.g., [^18^F]FDG] as well as a target-specific accumulating radiotracer ([^68^Ga]Ga-Fucoidan).

After manual PET data import and the user’s input, the data is automatically processed. Depending on the added free-to-chose anatomical atlas, the volume-of-interest (VOI) is selected. The segmentation of the VOI is done based on the atlas resized to the resolution of the PET images. The VOI, which is best aligned to the axes in the 3D space is chosen as reference for PET template creation. All other images are then registered onto the PET template. After alignment of the PET images to the anatomical atlas, the PET images can be evaluated by various semi-quantitative parameters. The evaluation as well as the included options for statistical analysis of the data are thereby defined by the sub-regions of the added anatomical atlas. Depending on the number of data per experimental group, the NU_DPA tool chooses either parametric or non-parametric tests.

The tool itself is based on Matlab code without a graphical user interface. This might be less user-friendly, but also offers more possibilities to easily adapt the code for the data evaluation with regard to own medical questions. The code also includes various Matlab-implemented algorithms, such as the *imresize3.m* function to adapt the image’s resolution. These pre-implemented codes should help to minimize the susceptibility to errors and to strengthen the validity of the tool. The tool is divided into several Matlab scripts including individual processing steps. This should help the user to adapt the code (if necessary), as well as it provides the possibility to validate different intermediate steps. An example would be the visual review of the results of (co-)registration processes.

Nuclear medicine Data Processing Analysis tool has been implemented using mono-modality PET data. Nevertheless, the registration algorithms included in this tool are also capable in registering multimodal data, e.g., PET-CT or PET-MR. For multimodal data, the segmentation and registration processes could therefore be further optimized. Similar to Deleye et al. (2017), data alignment could then be performed on the basis of the high-resolution anatomical data. The functional data could then be aligned using the resulting transformation matrices. However, possible necessary adjustments of the codes cannot be excluded in this regard. In this context, the tool should also be tested for the accuracy of the registrations, including the quantification of alignment errors. The respective evaluation of alignment or registration errors were beyond the scope of the present work. With the support of high-resolution anatomical image data, known positions contained in the image can be better identified.

In this tool, the anatomical atlas can be chosen independently by the user. It is therefore based on the actual structure of the volume-of-interest and not on a geometric body like e.g., in AMIDE (Loening and Gambhir, 2003). Though it has not been tested yet, this means that NU_DPA tool might also work for other organs or species than the analyzed stroke-related aspects in rat brain (e.g., heart). However, further tests in this regard are still pending. Of course, the option to add an atlas has the disadvantage of finding a suitable atlas, but it also has some advantages: On the one hand, depending on the availability of the atlas, further costs can be reduced. On the other hand, the atlas can be modified with regard to one’s own medical questions, e.g., by subdividing the brain into left and right hemispheres. Also, a possibly more suitable or recent atlas can be used instead of a pre-implemented atlas.

The commercial PMOD as well as the spmratIHEP Toolbox and SAMIT (Nie et al., 2014; Garcia et al., 2015), include pre-defined anatomical atlases. Individual adaptations are therefore difficult to implement. In the study of Deleye et al. (2017) for example, they analyzed multiple [^18^F]-labeled radiotracers in mice using PMOD. In order to add anatomical information, the additionally acquired CT datasets had to be registered on an atlas based on [^18^F]FDG-PET/CT data. That means, that functional data alone would not be sufficient to align the data with the atlas. Additionally, the calculated SUV of the data had to be corrected for blood glucose levels.

In comparison to the other data analysis tools mentioned above, which focus on the SUV, data can be evaluated by three semi-quantitative parameters (A_*N*_, SUV, and UR). This allows for a more flexible and comprehensive interpretation. For example with regard to test new types of radiotracers a temporal evaluation according to A_*N*_ can be helpful. It may provide information about the period in which the highest percentage tracer enrichment took place at a specific target *in vivo*. Furthermore, in studies in which radiotracer uptake is symmetrically distributed [e.g., [^18^F]FDG in brain], data evaluation with UR may be advantageous over SUV because UR considers only the actual radiotracer accumulated.

Similar to SAMIT, the NU_DPA is capable to create own data templates on which the acquired images are then registered onto. However, opposite to SAMIT, the creation of the templates is performed automatically without the need of choosing the reference data set by the user. Although it has been shown from the data listed that the NU_DPA tool is suitable for various radiotracers (unlike spmratIHEP Toolbox), further testing is required. This includes testing whether the tool can also be used, e.g., for SPECT radiotracers.

In contrast to the mentioned non-commercial data analysis tools (Loening and Gambhir, 2003; Nie et al., 2014; Garcia et al., 2015), NU_DPA also offers options to statistically analyze the acquired data. Thus, no further software program is needed to evaluate the data. The statistical tests are thereby based on Matlab-implemented algorithms (e.g., *friedman.m*). However, due to the data used to establish the tool, the tool is currently only tested and validated for the animal data mentioned in section “Animal Data.” This means, the focus was based on stroke-related neurological diseases, including small numbers of animals per experimental group (*n* < 30). Therefore, nonparametric tests were used in our studies to perform statistical data analysis. Additional data testing is needed to further improve the stability of the tool. Furthermore, the implemented parametrical statistical tests would still needed to be tested with larger, normal distributed datasets.

## Conclusion

Our semi-automatic, preclinical PET image data analysis tool NU_DPA offers a good alternative for analyzing functional processes *in vivo*. It is based on MRI templates and allows the creation of own PET templates on which the own acquired data will be registered onto. Furthermore, it offers the possibility to evaluate the acquired data by more than the mainly used semi-quantitative parameter SUV and also includes options for statistical data analysis. Based on the listed radiotracers ([^18^F]FDG, [^68^Ga]Ga-Fucoidan), it was demonstrated that the tool is potentially suitable for both metabolic radiotracers as well as target-selective radiotracers. To the best of our knowledge, this tool is so far the only one that allows such a “compact” data analysis of functional PET brain images of small animals.

## Data Availability Statement

The raw data supporting the conclusions of this article will be made available by the authors, without undue reservation.

## Ethics Statement

The animal studies were reviewed and approved by the District government of Lower Franconia.

## Author Contributions

FS implemented the NU_DPA tool and performed data analysis. FS, II, and SS provided revisions and approved the final version of the manuscript. All authors contributed to the article and approved the submitted version.

## Conflict of Interest

The authors declare that the research was conducted in the absence of any commercial or financial relationships that could be construed as a potential conflict of interest.

## Abbreviations

A_*N*_: normalized activity
[^18^F]FDG: 2-[^18^F]Fluoro-2-deoxyglucose
MIRT: medical image registration toolbox
MRI: magnet resonance imaging
NU_DPA: nuclear medicine Data Processing Analysis tool
OSEM2D: 2D-ordered subsets expectation maximization
OSEM3D: 3D-ordered subsets expectation maximization
PET: positron emission tomography
PT: photothrombosis
SPECT: single photon emission computed tomography
SUV: standardized-uptake-value
UR: uptake ratio
VOI: volume-of-interest.
